# Effect of Deformed Prior Austenite Characteristics on Reverse Phase Transformation and Deformation Behavior of High-Strength Medium-Mn Steel

**DOI:** 10.3390/ma17225618

**Published:** 2024-11-17

**Authors:** Ying Dong, Jingwen Zhang, Tao Liu, Mingxing Ma, Lei Zhu, Chengjun Zhu, Linxiu Du

**Affiliations:** 1 Henan Material Forming Equipment Intelligent Technology Engineering Research Center, Henan Polytechnic Institute, Nanyang 473000, China; zjwchris@163.com (J.Z.); zhchj222@sina.com (C.Z.); 2 State Key Laboratory of Rolling and Automation, Northeastern University, Shenyang 110819, China; 18340432177@163.com (T.L.); dulx@ral.neu.edu.cn (L.D.); 3 Key Laboratory of High Performance Metal Materials, Henan Polytechnic Institute, Nanyang 473000, China; manager92@163.com (M.M.); hnpizhu@163.com (L.Z.)

**Keywords:** prior austenite, reverse phase transformation, retained austenite, thermodynamics and kinetics, medium-Mn steel

## Abstract

In this study, microstructure evolution during prior austenite decomposition and reverse phase transformation processes was revealed in a high-strength medium-Mn steel. Furthermore, the relationship between deformed prior austenite characteristics and deformation behavior was studied. The results indicated that the recovery and recrystallization of the deformed prior austenite were significantly inhibited during hot rolling in the non-recrystallized zone, the grain size was obviously refined along the normal direction (ND), and that the strain hardening of prior austenite via hot deformation could increase the resistance of shear transformation, resulting in the preservation of high-density lattice defects in the quenched martensite matrix. Before the nucleation of intercritical austenite, the dislocation and grain boundary can provide fast diffusion paths for C and Mn, and the enrichment of C and Mn before intercritical austenite formation can reduce the critical temperature of ferrite/austenite transformation. The nucleated sites and driving force for intercritical austenite were strongly increased by rolling in the non-recrystallization region. The resistance of crack propagation was found to be enhanced by the sustained transformation-induced plasticity (TRIP) effect (via retained austenite with different stability) and for the laminated microstructure, the optimum properties were obtained as being a combination of yield strength of 748 MPa, tensile strength of 952 MPa, and total elongation of 26.2%.

## 1. Introduction

Intercritically annealed medium-Mn steels with 3–10 wt.% Mn (improving the thermal and mechanical stability of austenite) have been strong candidates for third-generation advanced steels, which hold promising application potential in automotive, marine, offshore platform, and engineering machinery due to their prominent advantages in excellent mechanical properties and industrial production reliability [1,2,3]. To achieve a superior combination of strength and ductility, a certain amount of retained austenite is usually introduced into the matrix, and the meta-stable austenite can be provoked to transform into martensite under the action of applied stress/strain, retarding plastic instability and necking fractures. Therefore, the common process steps for the preparation are basically as simple as obtaining ferrite (i.e., heavily tempered martensite, α) and intercritical austenite (γ) and combining them, as referred to austenite reversion treatment (ART) [4,5]. The phase transformation process is accompanied by element diffusion, and the stability of intercritical austenite is improved by the partitioning of the austenite stabilizer. During the subsequent cooling stage, the C- and Mn-enriched austenite phase can be either retained (the desired retained austenite) or partially transformed into fresh martensite [6,7,8].

In medium-Mn steels, the fraction and stability of retained austenite and the characteristics of the α matrix (ferrite or ferrite–martensite) play the key roles in the realization of excellent mechanical properties. Thus, the chemical composition design and thermomechanical controlled processing (TMCP) control usually revolve around tailoring the grain size, phase proportion, morphology, and internal structure characteristics, which are considered to be the main factors affecting deformation behavior [4,9]. With the improvement of TMCP technology, the precise control of microstructures has been developed rapidly. Recently, several studies on delamination strengthening in ultra-high strength steels have been reported. Shang et al. [10] proposed a design of heterogeneous lamellar microstructure in a body-centered cubic structural metal. The microstructure can promote the occurrence of delamination parallel to the direction of main crack propagation, reducing the effective stress at the crack tip, and an excellent toughness of impact energy exceeding 100 J at −80 °C was obtained. Chandan et al. [11] carried out a warm rolling process (laminated microstructure) of medium-Mn steel and found that the laminated microstructure was tailored by introducing a higher density of lattice defects in the ferrite and retained austenite phases, resulting in a simultaneous improvement in tensile properties. Sahoo et al. [12] showed a heterogenous lamellar microstructure consisting of ferrite and austenite; the sustained strain hardening introduced by retained austenite through the course of deformation led to an extraordinary strength–ductility combination. Hu et al. [13] conducted a direct intercritical rolling process on medium Mn steel, and a laminated microstructure consisting of thin δ-ferrite, retained austenite, and recrystallized ferrite was obtained. The product achieved strength and elongation of 46.1 GPa%. Inspired by the above, the preparation process for obtaining lamellar microstructure provides guidance for the fabrication of medium-Mn steels via the hot-rolling process. In fact, the characteristic of deformed prior austenite during hot deformation also has a significant impact on the tailor of the laminated substructure. It is meaningful to study the involved reverse phase transformation mechanism and deformation behavior related to the hot-rolling process.

In this study, the prior austenite with two different types of recrystallization or non-recrystallization were prepared via the hot-rolling process. Systematic studies on austenite nucleation, element diffusion, and interface migration behavior during ART were conducted. The influence of element content and diffusion rate on the thermodynamics and kinetics of intercritical austenite formation were discussed. Particular attention was paid to the relationship between prior austenite characteristics and microstructure evolution and deformation behavior.

## 2. Experimental Procedures

### 2.1. Material and Thermomechanical Processing

The experimental steel was melted in a vacuum induction furnace and cast as 155 kg ingots with 60 mm thickness. The chemical composition and critical phase transformation temperatures (calculated by Thermo-Calc software 2023) of the experimental steel are listed in Table 1.

**Figure 1:**
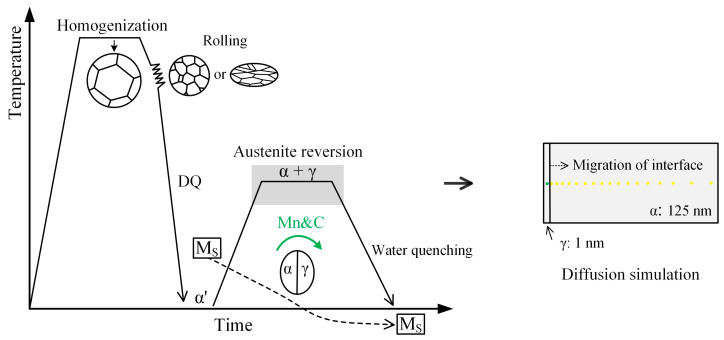
Schematic illustrations of thermomechanical controlled processes and heat treatment processes and initial conditions of diffusion simulation.

The slabs were reheated to 1200 °C and kept for 3 h for homogenization, then air cooled to the rolling temperature. After that, they were hot rolled into 6 mm thick strips; the total reduction was 88.3%. Then, the strips were directly water quenched (WQ) to the ambient temperature, with a cooling rate of ~50 °C/s. The finished rolling temperature of 1000 °C in the recrystallization region (R1000) and 820 °C in the non-recrystallization region (R820) were selected to obtain different types of prior austenite. The WQ strips were subsequently annealed at the α + γ phase region and kept for 0.5 h for intercritical austenite formation, then water quenched to the ambient temperature. Annealing temperatures of 570 °C (lower than the α + γ phase region), 610 °C (slightly lower than the α + γ region), and 630 °C (α + γ phase region) were selected to study the transformation kinetics and thermodynamic characteristics of intercritical austenite. The schematic illustrations of TMCP and heat treatment processes and the initial conditions of diffusion simulation are shown in Figure 1. For simplicity, the preparation process parameters are given in Table 2.

### 2.2. Simulation Condition of Thermodynamic and Kinetics

The simulation calculation of thermodynamics and kinetics for phase transformation was completed with Thermo-Calc software, using TCFE9 v9.3 and MOBFE4 v4.0 databases. The critical phase transformation temperature, phase volume fraction, and element content in FCC/BCC under equilibrium transformation were calculated. The Gibbs free energy (GFE) of ferrite and austenite for different steel grades was calculated to study the effect of C and Mn content on the critical phase transformation temperature. The element content in ferrite and austenite was fixed as seen in the chemical composition in Table 1 because of the nonequilibrium transformation in the practical preparation process (intercritical austenite formation during ART). The DICTRA module was used to explore the migration of phase transformation kinetics during intercritical austenite formation. To simplify the model, the austenite stabilizers (C and Mn) and the enriched elements in ferrite (Si and Al) were carried out under the simulation conditions. The planar mode with 100 grids in each phase unit was set to assume the phase growth morphology, and equal proportion spacing of 1.05 in austenite and 0.95 in ferrite was used, as shown in Figure 1.

### 2.3. Microstructure Characterization and Mechanical Properties Tests

The microstructural morphology and Mn element distribution were observed by optical microscope (OM, SUNNY, Hong Kong, China) and a JXA-8530F electron probe microanalyzer (EPMA, JEOL. Co., Ltd., Tokyo, Japan). The crystallographic information was obtained using a Gemini 300 scanning electron microscope (SEM, Carl Zeiss, Oberkochen, Germany) equipped with an electron backscattered diffractometer (EBSD). The lath width of martensite was measured by a Tecnai G2 F20 transmission electron microscope (TEM, FEI company, Hillsboro, OR, USA). The fracture surface of tensile samples was observed using a Zeiss Ultra 55 SEM (Carl Zeiss, Oberkochen, Germany). The volume fraction of retained austenite was measured by a Smart Lab 9 kW X-ray diffractometer (XRD, Rigaku, Tokyo, Japan) with a Cu Kα radiation source. For OM and EPMA observation, the samples were mechanically polished and etched with 4 vol% nital and 96 vol% CH_3_CH_2_OH. The EBSD and XRD samples were prepared via standard mechanical grinding and polishing procedures using a solution containing 6 vol% HClO_4_ and 94 vol% CH_3_CH_2_OH. The TEM sample was mechanically thinned to ~45 μm, and punched to round disks, then twin-jet polished using a solution containing 8 vol% perchloric acid and 92 vol% CH_3_CH_2_OH. The equations in the reference were used to measure the volume fractions of retained austenite [14].

The standard tensile samples with dimensions of 25 mm in gauge length and 2 mm in thickness were machined with a tensile axis parallel to the rolling direction (RD), and then tested at a crosshead speed of 3 mm/min using a SANS CMT5105 (SUNS Technology Stock CO., LTD, Shenzhen, China) universal testing machine at ambient temperature. Vickers hardness was measured using an MHV-1000 Z microhardness tester (Truer, Shanghai, China) at a 500 gf load for 10 s.

## 3. Results and Discussion

### 3.1. Effect of Deformed Prior Austenite Characteristics on Microstructure Evolution During Prior Austenite Decomposition and Reverse Phase Transformation Processes

**Figure 2:**
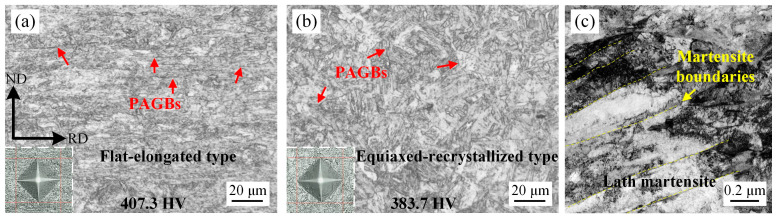
Micrograph of water-quenched experimental steels and hardness. (**a**) optical microscope (OM) micrograph of R820 sample; (**b**) OM micrograph of R1000 sample; (**c**) transmission electron microscope micrograph of R1000 sample.

The micrographs and hardness of the water-quenched experimental steels are shown in Figure 2. The prior austenite grain boundaries (PAGBs) are marked with red arrows. The prior austenite grain (PAG) in the R1000 sample exhibited the equiaxed-recrystallized type, which illustrated that the recovery and recrystallization occurred during the hot rolling process. A martensite matrix with an average lath width of ~242 nm was obtained, as shown in Figure 2c. For the R820 sample, the flat-elongated PAGs were obviously refined along the normal direction (ND), the recovery and recrystallization behavior of deformed austenite were significantly inhibited, and the strain hardening of deformed prior austenite increased the lattice resistance of martensite transformation, resulting in a lower martensite-start (M_s_) temperature [15]. Large amounts of lattice defects introduced by hot deformation were preserved in the quenched martensite. In addition, the size of the martensite was limited by the PAGBs. As a result, a refined martensite matrix with a higher density of lattice defects and higher hardness of 407.3 HV was obtained in the R820 sample.

**Figure 3:**
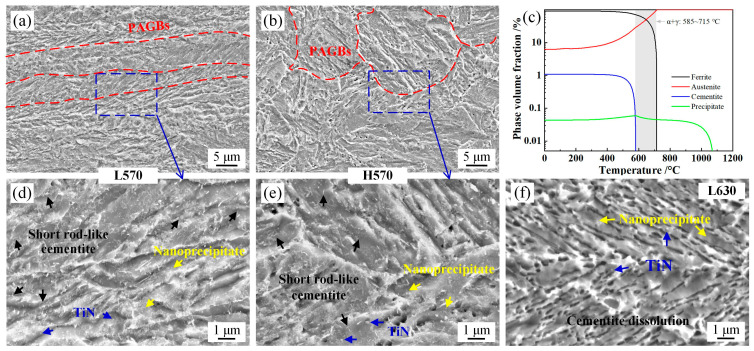
Scanning electron microscope (SEM) micrograph of experimental steels and equilibrium phase diagram. (**a**,**d**) L570 sample; (**b**,**e**) H570 sample; (**c**) equilibrium phase diagram; (**f**) L630 sample.

The SEM micrographs and equilibrium phase diagram are presented in Figure 3. The results show that the pancaked PAGs in the L570 sample retained the flat-elongated feature (laminate microstructure), the lath-like matrix structure exhibited distinct annealing characteristics, and large amounts of short rod-like cementite formed in the slow heating and holding process [16]. The quenched martensite underwent annealing recovery, in which the carbon diffused from supersaturated martensite and combined with the alloying element as a secondary phase. The utilization of Ti microalloying in the experimental steel resulted in rectangular-shaped TiN and nanoscale precipitates in the microstructure [17,18,19]. In contrast, the PAGs in the H570 sample displayed equiaxed-recrystallization features, and the length of the martensite lath was greater than in the L570 sample. The second phase contained cementite, TiN, and nanoscale precipitates, as shown in Figure 3b,e. As the annealing temperature increased to 630 °C (α + γ two-phase region), the cementite dissolved in the L630 sample, which was consistent with the equilibrium phase calculation result in Figure 3c. The Ti-containing precipitates were observed due to the higher thermal stability. The lath size of the matrix was increased due to active recrystallization. The blocky ferrite distributed along the PAGBs indicated that the martensite recrystallization and intercritical austenite formation occurred simultaneously at lattice defect-rich areas [20].

**Figure 4:**
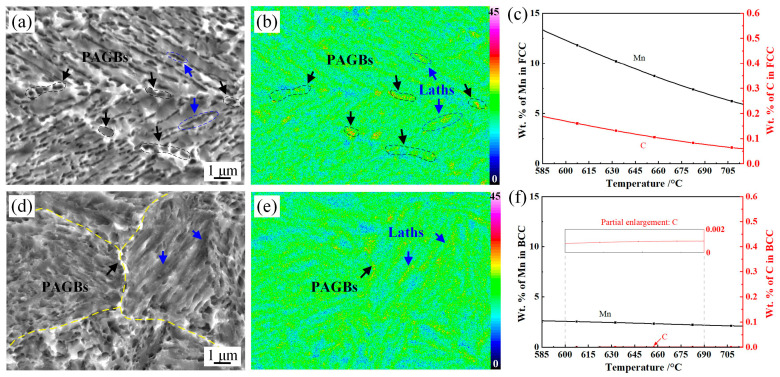
SEM micrograph, Mn distribution, and the corresponding equilibrium calculation of experimental steels. (**a**) SEM micrograph of L630 sample; (**d**) SEM micrograph of H630 sample, the prior austenite grain boundaries were marked with the yellow dotted lines; (**b**,**e**) the corresponding Mn distribution; (**c**) Mn and C content in FCC; (**f**) Mn and C content in BCC.

The formation of a new phase and the recovery/recrystallization of the parent phase occurred simultaneously during the ART process, leading to significant element partitioning behavior. The Mn-rich regions are observed both at the flat-elongated PAGBs and the lath boundaries in the L630 sample, as shown in Figure 4a,b. The equilibrium calculation results indicate that the solubility of C and Mn in austenite was significantly higher than that in ferrite, exhibiting a pronounced tendency for diffusion from ferrite to austenite in the α + γ phase region. For example, the Mn content in austenite at 630 °C was 10.36 wt.%, which was 4.2 times higher than that in ferrite. Mn is a well-known austenite stabilizer in medium-Mn steels; thus, the Mn-rich regions were intercritical austenite during the holding process in the α + γ phase region. The blocky intercritical austenite was most discretely distributed along the PAGBs, while the lath intercritical austenite was mainly formed along the tempered martensite boundaries. This is due to the fact that the enriched lattice defects at the PAGBs provided sufficient nucleation sites and acted as a driving force for intercritical austenite formation [21]. In the H630 sample, the grain size and Mn content of the intercritical austenite were decreased, which was due to the fact that the lattice defect density in the pre-annealing martensitic matrix and PAGBs was reduced by the active recrystallization of the deformed prior austenite during hot rolling. As the grain size and homogenization of austenite stabilizers increased, part of the intercritical austenite with poor thermal stability transformed into fresh martensite during the subsequent water-quenching process [22].

**Figure 5:**
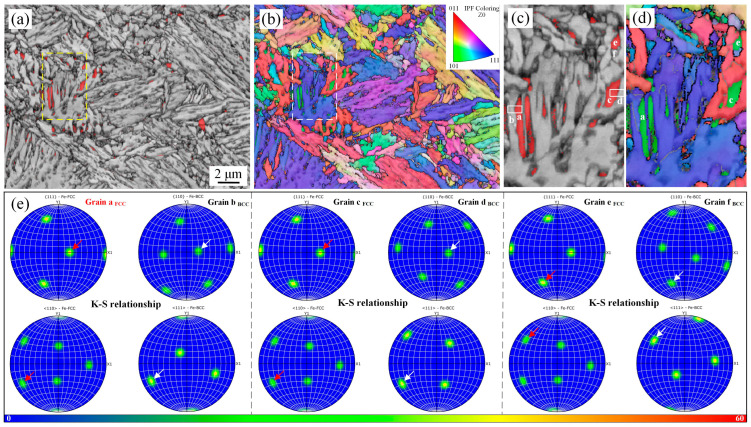
Microstructure of H630 sample analyzed by electron backscattered diffractometer (EBSD). (**a**) Combined map of band contrast image and phase image; (**b**) Inverse pole figure; (**c**,**d**) the enlargement of the corresponding squares region in (**a**,**b**); (**e**) crystallographic analysis of several grains, which are pointed out as a, b, c, d, e, and f in Figure 6c, the arrows represent the specific orientation.

Figure 5 shows the EBSD results of the H630 sample. The retained austenite phase is colored in red. Both blocky and lath-like retained austenite can be observed in Figure 5a. Blocky retained austenite was mainly distributed along the PAGBs and packet boundaries, while lath-like retained austenite was located at the tempered martensite laths. The rectangular region in Figure 5a was used to study the crystallographic relationship between retained austenite and neighboring ferrite and martensite. According to the IPF image and pole figure, the retained austenite distributed between the ferrite laths followed a specific orientation relationship with the parent phase. This is due to the fact that the newly formed intercritical austenite showed the original orientation of the PAGs upon reversion without α’ recrystallization, and the lath-like ferrite (heavily tempered martensite) was transformed from the recovery and element repartition of quenched martensite without recrystallization [23]. Thus, the retained austenite ‘a’ and ‘c’ followed the Kurdjumov-Sachs (K-S) orientation relationship with ferrite ‘b’ and ‘d’, respectively, i.e., shape memory effect. Additionally, the austenite ‘e’ followed the K-S orientation relationship with grain ‘f’. Based on the morphology, it can be inferred that the grains stemmed from the same single intercritical austenite before austenite decomposition. The large-sized austenite with poor thermal stability partially transformed into martensite during the water-quenching process.

### 3.2. The Kinetics and Thermodynamics of Intercritical Austenite Formation in the Experimental Steel with Different Characteristics of Deformed Prior Austenite

The austenite formation under the reverse transformation in this study underwent a diffusional mechanism due to the slow heating rate. Diffusion paths, annealing temperature, and time are the main factors that determine intercritical austenite formation [4,24]. To study the effect of element diffusion on microstructure evolution during the ART process, the GFE of austenite and ferrite for different steel grades was calculated, as shown in Table 3. Under constant temperature and pressure, the spontaneous phase transformation occurs as the ΔG ˂ 0 (G_new phase_ − G_parent phase_). The critical phase transformation temperature is T_ΔG=0_. The results show that the ΔG_FCC-BCC_ decreased with an increase in temperature, and the T_ΔG=0_ was close to 610 °C. However, the α + γ phase region was 585–715 °C using the Property Diagram module, as shown in Figure 3c. The underlying reason for this is that the element was partitioned between austenite and ferrite in the α + γ phase region, as evidenced in Figure 4c,f. Two types of steel, 0.6C-5.8Mn steel (high C content) and 0.06C-10Mn (high Mn content) steel, were selected to study the change in GFE with different C and Mn content. It was found that ΔG decreased with an increase in C and Mn content, i.e., the enrichment of Mn and C before austenite formation could reduce the critical temperature of ferrite to austenite transformation during the ART process.

**Figure 6:**
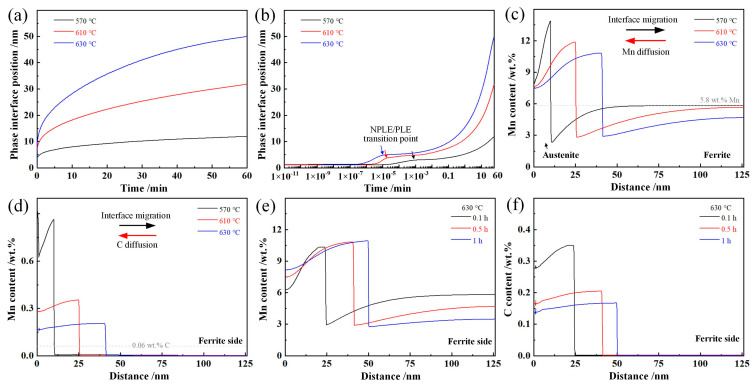
DICTRA calculation results of austenite/ferrite interface migration and element diffusion during intercritical austenite growth process. (**a**,**b**) Phase interface position versus isothermal duration; (**c**,**d**) Mn/C content profiles after isothermal holding at different temperatures for 0.5 h; (**e**,**f**) Mn/C content profiles after isothermal holding at 650 for different duration.

The DICTRA calculation results of phase interface migration and element diffusion under local equilibrium are shown in Figure 6. The thickness of austenite lath was increased proportionately with the increasing isothermal duration in the simulation time. The migration distance of austenite/ferrite interface after isothermal holding for 0.5 h was 10.2 nm (570 °C), 25.4nm (610 °C), and 41.0 nm (630 °C), respectively. It was interesting that the austenite growth was sustained even when the holding temperature (570 °C) was lower than the T_ΔG=0_ (~610 °C). The inflection points in Figure 6b represent the transition between negligible-partitioned local equilibrium (NPLE) mode and partitioned local equilibrium (PLE) mode. In the initial phase transformation stage before these points, the kinetics of phase interface migration were controlled by speedy C diffusion. Then, the growth of austenite was controlled by interfacial diffusion of the alloying element [25,26]. The transition time was increased with the decrease in isothermal temperature due to the decreasing diffusion rate. Figure 6c,d shows the evolution of Mn and C profiles during the isothermal process. The migration direction of the phase interface is opposite to the diffusion direction of Mn/C elements. The austenite stabilizers were diffused from ferrite to the interface, and the growth of austenite was realized through the migration of the α-γ boundary toward the ferrite side. Thus, the growth rate of intercritical austenite during the ART process depended on the element diffusion rate in ferrite [27,28]. The thickness of austenite increased with an increase in isothermal temperature, and the average element content of Mn and C also decreased, which indicated that increasing the isothermal temperature would result in more intercritical austenite, decreasing its average Mn and C content. The isothermal duration is expected to have a similar effect to the isothermal temperature, as shown in Figure 6e,f [29,30]. The simulation calculation results also prove that the stability in a single intercritical austenite at the different regions was not uniform due to the heterogeneous Mn and C distribution. The martensite would form at the austenite region with poor thermal stability during the water-quenching process.

**Figure 7:**
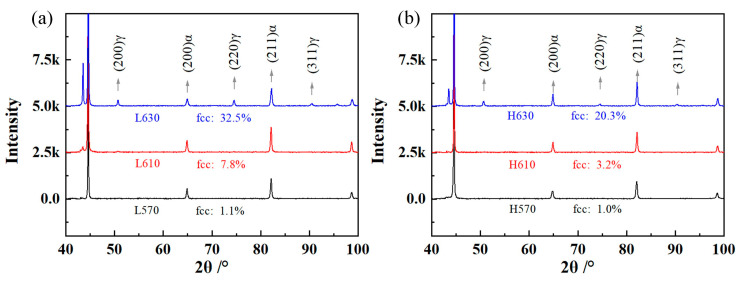
X-ray diffractometer patterns of experimental steels. (**a**) L570, L610, and L630 samples; (**b**) H570, H610, and H630 samples.

The XRD patterns of annealed samples are shown in Figure 7. For the L610 sample, the ΔG_FCC-BCC_ was 3.4 kJ/mol > 0; however, retained austenite at 7.8 vol.% in the L610 sample was obtained. The underlying reason for this is that the starting microstructure prior to ART isothermal holding was quenched martensite. The C/Mn content in the quenched martensite was supersaturated (as shown in Figure 3), which exhibited a pronounced tendency for diffusion from martensite to the lattice defects before austenite formation. For the BCC phase, the dislocation pipe diffusion, grain boundary diffusion, GB migration, and recovery of dislocations (moving dislocation pipes) aided the solute diffusion of Mn/C [21]. Large amounts of lattice defects and deformation energy introduced by the low-temperature hot deformation were preserved in the quenched martensite, which provided the driving force for Mn/C diffusion from the supersaturated BCC phase to the lattice defects (dislocation, PAGB, and phase interface) before intercritical austenite formation. The critical temperature for austenite formation in the Mn- and C-enriched regions was decreased, as evidenced in Table 3. The lattice defects could provide fast diffusion paths for Mn/C diffusion. For the H610 sample rolled in the recrystallization region, the distortional strain energy and lattice defects were partially annihilated due to the recovery and recrystallization of PAGs, which led to delaying the austenite formation during the annealing process. For the steels annealed at 570 °C, the ΔG_FCC-BCC_ was 168.0 kJ/mol. The decrease in ΔG through the diffusion of Mn and C overcame the phase transformation barrier, then the nucleation of intercritical austenite occurred [31,32]. The nucleation and growth rate of austenite were slow due to the lower diffusion coefficient at 570 °C. For the samples annealed at higher temperatures, the fractions of retained austenite were 32.5% and 20.3%, respectively, in samples L630 and H630. Combined with the equilibrium calculation results in Figure 3c and Figure 6, the phase transformation behavior has not yet reached the equilibrium state.

### 3.3. Effect of Deformed Prior Austenite Characteristics on Deformation Behavior

The tensile properties and volume fraction of retained austenite (V_RA_) are provided in Table 4. The R_p0.2_ strength was determined as the yield strength (YS) because of the continuous yield behavior. The YS of annealed samples decreased with an increase in ART temperature due to the active recovery/recrystallization of quenched martensite and the increasing volume fraction of retained austenite. The resistance of deformation dislocation slip was decreased during the initial tensile deformation stage. However, the effect of annealing temperature on ultimate tensile strength (UTS) between the steels with different characteristics of prior austenite was different. The UTS was increased with decreasing ART temperature in the steels with equiaxed-recrystallized PAGs. The UTS decreased by 50 MPa as the ART temperature was increased from 570 °C to 630 °C. For the steels with flat-elongated PAGs, the UTS was decreased first and then increased.

**Figure 8:**
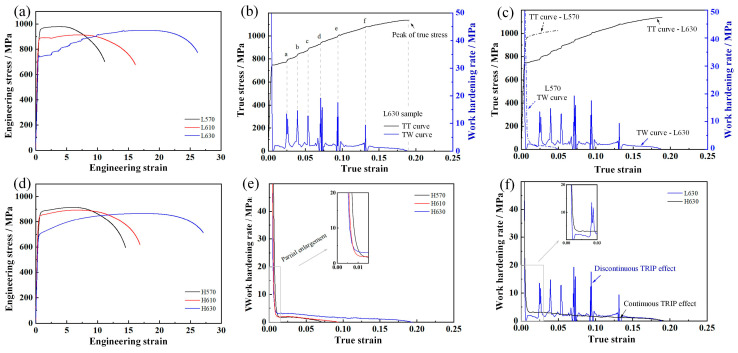
Engineering stress–strain curves, true strain–stress (TT), and true strain–work hardening rate (TW) curves of experimental steels. (**a**) Engineering stress-strain curves of samples with flat–elongated PAGs; (**b**) combined TW and TT curve of L630 sample, the letters a–f represent the sudden change points; (**c**) combined TT and TW curves of samples L570 and L630; (**d**) engineering stress-strain curves of samples with equiaxed-recrystallized PAGs; (**e**) TW of samples with equiaxed-recrystallized PAGs; (**f**) TW curves of samples L630 and H630.

Figure 8 shows the engineering strain–stress curves, true strain–stress (TT) curves, and work hardening rate (TW) curves of annealed samples. The deformation resistance and work hardening behavior of samples with different deformed prior austenite characteristics are obviously different. The serrated fluctuations in tensile behavior for the L630 sample was observed, and the work hardening capacity was improved by the TRIP effect, as shown in Figure 8a,b. The tensile deformation behavior of L570 (almost fully ferrite phase) and L630 (32.5 vol.% retained austenite) are shown in Figure 8c. In the micro strain zone (elastic deformation), both samples exhibited high work hardening values due to the resistance of dislocation slip. With the accumulation of deformation, the work hardening rates were decreased according to the combined effect of work hardening (by dislocation tangle and multiplication) and softening (by stress relaxation and dislocation glide). The comparison results show that the L570 sample exhibited a relatively obvious work hardening effect by the dislocation interaction in this tensile stage. This was due to the significant recovery/recrystallization of the martensitic matrix and austenite formation in the L630 sample (relatively lower YS value). As the deformation increased to the critical value, the TRIP effect of retained austenite occurred, which improved the work hardening ability. The upward curvature of the work hardening curves in Figure 8c shows clear evidence of the activation of the TRIP effect. Good ductility with total elongation of 26.2% was obtained in the L630 sample due to the sustained TRIP effect over a large strain range.

The TW of samples with equiaxed-recrystallized PAGs are shown in Figure 8e. The TRIP effect of retained austenite was firstly activated in sample H630, and the work hardening effect was more significant than H570 and H620 samples, exhibiting a lower yield ratio. Combined with the curves shown in Figure 8d, the softening effect by recovery/recrystallization of martensitic matrix and austenite formation was higher than the hardening effect via the TRIP effect with an increase in isothermal temperature, which led to decreases in YS and UTS. The TW curves of samples with different prior austenite characteristics are shown in Figure 8f, and the tensile behavior of the two samples was obviously different. For the L630 sample with a discontinuous TRIP effect, the work hardening rate remained at a low value at the initial plastic deformation stage (ε < 0.023), which indicated that the mechanical stability of retained austenite in the L630 sample was relatively higher than that in the H630 sample. The lattice defects introduced by low-temperature rolling could provide the fast diffusion paths for Mn/C diffusion during the intercritical austenite formation, which enhanced the driving force and nucleation site density for austenite reversion transformation. It should be emphasized that the increase in dislocation density within austenite had minimal impact on work hardening, with the primary contribution coming from the BCC structure. Austenite itself did not provide significant hardening, but the accumulation of dislocations within the austenite phase facilitated more martensitic transformation, which in turn enhanced hardening [20]. Once the dislocation density in austenite reached a critical level, the hardening induced by the TRIP effect outstripped local softening, leading to the finish of yield point elongation. The serrated fluctuations in tensile behavior were caused by the discontinuous TRIP effect (retained austenite with different mechanical stability). As a result, optimum properties comprising a combination of YS of 748 MPa, UTS of 952 MPa, and TEL of 26.2% were obtained in L630 sample, which was derived from the contribution of the sustained TRIP effect of austenite and the cooperative deformation of ferrite. For the H630 sample, a smooth engineering strain–stress curve was obtained due to the continuous TRIP effect.

**Figure 9:**
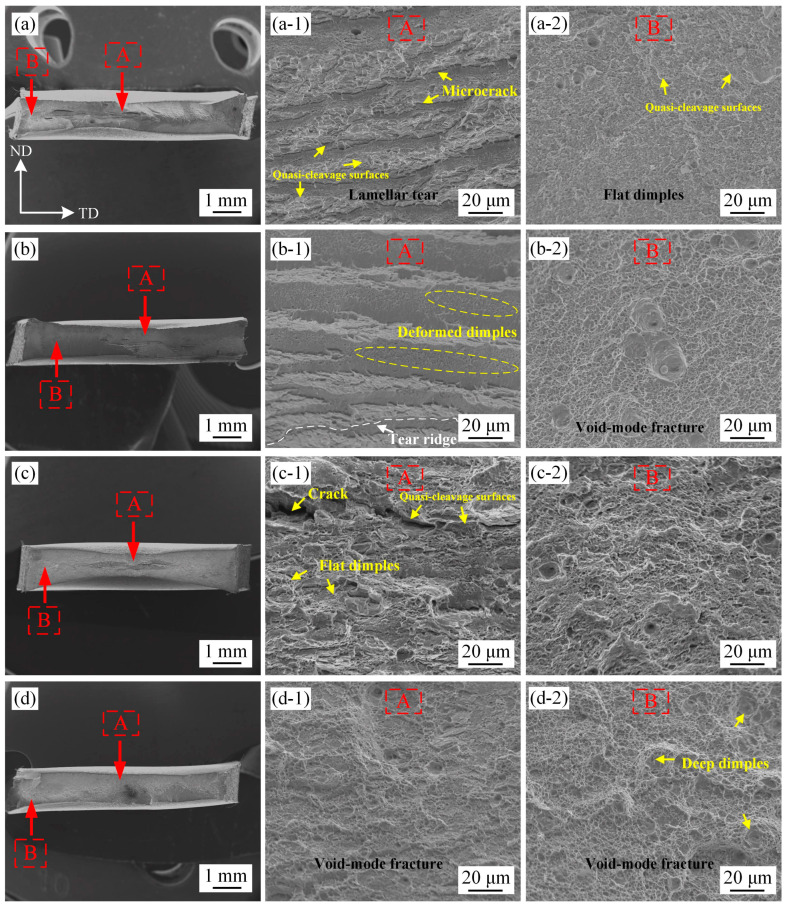
Fracture morphology of tensile samples after being subjected to different processes. Zone A represents the rapid crack propagation zone, zone B represents the plastic deformation concentration zone. (**a**) L570; (**b**) L630; (**c**) H570; (**d**) H630; (**a-1**,**b-1**,**c-1**,**d-1**) zone A; (**a-2**,**b-2**,**c-2**,**d-2**) zone B.

The fracture morphology of tensile samples is shown in Figure 9, whereby the rapid crack propagation zone and plastic deformation concentration zone are represented as zones A and B, respectively. In the L570 sample with an almost fully ferrite phase, zone A showed a typical lamellar tear fracture, which contained microcrack and quasi-cleavage surfaces. Meanwhile, flat dimples and a few small quasi-cleavage surfaces were observed in zone B, and the weak crack arrest ability could not consume more energy during the crack propagation period, exhibiting a poor plasticity with TEL of 11.2%. In the L630 sample with 32.5 vol.% retained austenite, zone A was characterized by a lamellar tear with a deformed dimples fracture. The plastic deformation was more obvious and deeper dimples were found in zone B. These dimples can absorb more deformation energy than quasi-cleavage surfaces and tear ridges during the rapid crack propagation period, resulting in a higher plasticity with a TEL of 26.2%. For the samples with equiaxed-recrystallized PAGs, some large microcracks were found near the cleavage surfaces in zone A of the H570 sample, and the fractures also contained some flat dimples. Zone B was composed of relatively larger and deeper dimples than in the L570 sample, which can consume more deformation energy. The H630 sample showed void-mode fracture in zones A and B; however, overall, the microstructure was more uniform than in the L630 sample, and no lamellar fractures appeared. The laminated substructure of experimental steel was determined by the characteristics of the deformed prior austenite and ART process.

## 4. Conclusions

The significant role of deformed prior austenite on microstructure evolution during prior austenite decomposition and reverse phase transformation processes was investigated in high-strength medium-Mn steel. The mechanisms of microstructure evolution and tensile behavior were discussed, and the main conclusions are as follows:

(1) The recovery and recrystallization behavior of deformed prior austenite are significantly inhibited during rolling in the non-recrystallized zone. The morphology of PAGs changed from equiaxed type (rolling in the recrystallization region) to flat-elongated type (rolling in the non-recrystallization region). A refined martensite lath with high-density lattice defects can be obtained via the strain hardening of prior austenite.
(2) The blocky intercritical austenite is mainly formed at PAGBs and packet boundaries with a high density of lattice defects, where the recrystallization of ferrite and the formation of intercritical austenite occur simultaneously. The lath-like RA is mainly formed along boundaries of lath-like ferrite with incomplete recovery. The strain hardening of deformed prior austenite by rolling in the non-recrystallization region can decrease lattice defects in the quenched martensite matrix.
(3) The ΔG^FCC-BCC^ is decreased with an increase in temperature and C/Mn content. The enrichment of C/Mn before intercritical austenite nucleation can reduce the critical temperature of ferrite to austenite transformation during the ART process. The dislocation and grain boundary can provide fast diffusion paths for C and Mn. The nucleation and growth of intercritical austenite are accelerated by high-density lattice defects and refined martensitic lath in the sample with flat-elongated PAGs.
(4) The tensile deformation resistance is dependent on the work hardening effect (via TRIP effect and dislocation tangle and multiplication) and softening effect (via stress relaxation and dislocation glide). The optimum properties were obtained in the L630 sample, with a combination of yield strength of 748 MPa, tensile strength of 952 MPa, and total elongation of 26.2%, mainly attributed to the sustained TRIP effect and the laminated microstructure.

## Figures and Tables

**Table 1:** Chemical composition (wt.%) and critical phase transformation temperatures (°C).

C	Mn	Si	Al	Ti	N	Cr + Ni + Mo + Cu	Fe	A_e1_	A_e3_
0.06	5.8	0.2	0.02	0.02	0.004	Trace	Bal.	585	715

**Table 2:** Parameters of preparation processes of experimental steels.

Steel No.	Finish Rolling Temperature/°C	Annealing Temperature/°C
L570	820	570
L610	820	610
L650	820	650
H570	1000	570
H610	1000	610
H650	1000	650

**Table 3:** GFE calculation for different steel grades with different C and Mn contents (J/mol).

Temperature ^(a)^	0.06C-5.8Mn Steel			0.6C-5.8Mn Steel			0.06C-10Mn Steel		
	G_BCC_	G_FCC_	ΔG	G_BCC_	G_FCC_	ΔG	G_BCC_	G_FCC_	ΔG
570 °C	−34,678.3	−34,510.3	168.0	−33,786.5	−33,978.6	−192.1	−35,326.0	−35,608.2	−282.2
610 °C	−37,246.6	−37,243.2	3.4	−36,285.9	−36,710.9	−425.0	−37,992.4	−38,375.1	−382.7
630 °C	−38,565.6	−38,631.2	−65.6	−37,569.2	−38,099.4	−530.2	−39,362.6	−39,780.3	−417.7

^(a)^ P.S. 0.06C-5.8Mn steel: Experimental steel; ΔG = ΔG_FCC-BCC_ = G_FCC_-G_BCC_, kJ/mol.

**Table 4:** Tensile properties and volume fraction of retained austenite of experimental steels.

Steel No.	YS/MPa	UTS/MPa	Yield Ratio	TEL/%	V_RA_/%
L630	748 ± 3	952 ± 7	0.79	26.2	32.5
L610	875 ± 5	898 ± 3	0.97	16.0	7.8
L570	948 ± 8	970 ± 12	0.98	11.2	1.1
H630	697 ± 7	868 ± 10	0.80	27.0	20.3
H610	833 ± 3	880 ± 13	0.95	16.8	3.2
H570	837 ± 4	908± 8	0.92	14.5	1.0

## Data Availability

The original contributions presented in the study are included in the article, further inquiries can be directed to the corresponding author.
